# Plant Flavonoid—Enriched Functional Foods for Polycystic Ovary Syndrome: Dietary Sources, Bioavailability Enhancement, and Personalized Intervention Strategies

**DOI:** 10.1002/fsn3.72350

**Published:** 2026-09-17

**Authors:** Jianding Wang, Hejian Zhu, Qing Shen, Yinghua Zhong, Yunli Du, Wenxi Yu, Fang Mao, Zhitao Yao, Weiwei Zhu, Lirong Sun, Qiu Lin, Xiaomin Xu, Jiong Zhu

**Affiliations:** ^1^ Key Laboratory for Traceability and Efficacy Research of Genuine Medicinal Materials Hangzhou Linping Hospital of Traditional Chinese Medicine Hangzhou Zhejiang China; ^2^ Laboratory of Food Nutrition and Clinical Research, Institute of Seafood Zhejiang Gongshang University Hangzhou China; ^3^ Quzhou Hospital of Traditional Chinese Medicine (Four‐Province Bordering Hospital), Zhejiang Chinese Medical University Quzhou Zhejiang China; ^4^ Panvascular Diseases Research Center The Quzhou Affiliated Hospital of Wenzhou Medical University, Quzhou People's Hospital Quzhou China

**Keywords:** bioavailability, dietary intervention, functional foods, plant flavonoids, polycystic ovary syndrome

## Abstract

Plant flavonoids are polyphenolic compounds widely found in fruits, vegetables, tea, legumes, and medicinal and edible plants; they are important natural functional components in food. These compounds play a significant role in various biological activities, such as alleviating inflammation, regulating insulin sensitivity, and improving glucose and lipid metabolism, making them ideal candidates for the development of functional foods and dietary intervention strategies aimed at balancing endocrine and metabolic functions. This paper first systematically reviews the dietary sources and distribution of plant flavonoids, their structural classification, and the factors determining their intestinal metabolism and bioavailability; it then summarizes the key molecular mechanisms and experimental studies regarding the intervention of plant flavonoids in polycystic ovary syndrome (PCOS). Building on this foundation, from the practical perspective of functional food development, the paper focuses on the application of modern food processing technologies—such as microencapsulation, nano‐encapsulation, liposomal carriers, enzymatic hydrolysis and fermentation, and synergistic effects with excipients—in enhancing the bioavailability of plant flavonoids. It analyzes technical approaches for regulating stability and sensory quality in formulation design and proposes practical dietary strategies and personalized nutrition plans for individuals with PCOS. Finally, future research directions for flavonoid dietary interventions are outlined, aiming to provide guidance for the research, development, and commercialization of functional foods containing plant flavonoids, and to promote the food industry's transition toward precision nutrition and personalized health interventions.

## Graphical abstract

Plant flavonoids from fruits, vegetables, tea, and soybeans require food processing technologies (microencapsulation, nano‐encapsulation, lipid carriers, fermentation, food collaboration) to enhance bioavailability, enabling the development of functional foods for precision dietary intervention in PCOS.

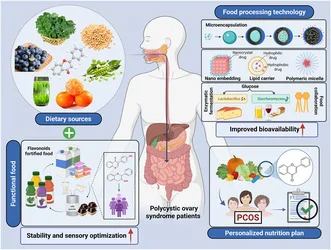

## 1 Introduction

Plant flavonoids, a class of polyphenolic compounds widely found in everyday foods such as fruits, tea, and vegetables, are important natural functional components in the human diet (Noorbakhsh Varnosfaderani et al. 2025). Based on differences in their parent ring structures and substituents, plant flavonoids can be classified into several subclasses, including flavones, flavanols, flavanones, isoflavones, and anthocyanins, each of which exhibits distinct distribution patterns across different food sources (Galatro et al. 2024; Zhao et al. 2024). Numerous studies have confirmed that plant flavonoids possess multiple biological activities, including antioxidant and anti‐inflammatory effects, as well as the ability to regulate glucose and lipid metabolism and improve insulin sensitivity. This makes the intake of flavonoid compounds through the daily diet a potential strategy for the prevention and adjunctive treatment of various chronic metabolic diseases (Armanini et al. 2022).

Polycystic ovary syndrome (PCOS) is one of the most common endocrine and metabolic disorders among women of childbearing age, with a global prevalence of approximately 5%–15%. It is characterized by hyperandrogenism, ovulatory dysfunction, and insulin resistance, and is closely associated with obesity and abnormalities in glucose and lipid metabolism (Jiang 2025; Meier 2018). Reproductive dysfunction in PCOS stems not only from ovulatory dysfunction but also involves defects in oocyte quality and abnormalities in endometrial receptivity, and is associated with a significantly increased risk of pregnancy complications (Palomba 2021; Palomba et al. 2021, 2017, 2015). These multiple pathological mechanisms, spanning from follicular development to pregnancy outcomes, provide multidimensional targets for dietary intervention. Currently, mainstream interventions for PCOS include oral contraceptives, metformin, and antiandrogens; while these can alleviate symptoms, long‐term use is associated with side effects, and relapse is common after discontinuation. Traditional Chinese medicine (TCM) syndrome differentiation and treatment, as well as acupuncture, are also employed, but their efficacy varies greatly among individuals (Alesi et al. 2023; Zhang et al. 2021). The limitations of these treatment approaches highlight the significant potential of dietary intervention as a complementary or alternative method for this complex condition.

Given these treatment limitations, dietary intervention—as a fundamental and sustainable adjunctive strategy—holds promise for providing a new pathway for the comprehensive management of PCOS and addressing the shortcomings of existing treatments. The daily consumption of flavonoid‐rich foods not only leverages the safety advantages of natural compounds but also, through “multicomponent, multi‐pathway” synergistic effects, exerts potential regulatory effects on multiple pathological aspects of PCOS—including hyperandrogenism, ovulatory dysfunction, and metabolic abnormalities—thereby providing a scientific basis and practical pathway for improving reproductive health and long‐term metabolic risks in PCOS patients through dietary intervention. Therefore, this review aims to provide a new perspective for researchers in the fields of food nutrition and dietary intervention, serve as a reference for clinicians in developing comprehensive diet‐based intervention plans, and further expand new pathways for PCOS patients to improve their reproductive health and metabolic risks through dietary adjustments.

It should be pointed out that the latest international PCOS management guidelines (2023 International Evidence Based Guidelines) emphasize that a healthy lifestyle should be the core of management for all PCOS women, but do not provide specific recommendations for specific dietary patterns or micronutrient supplementation programs (Teede et al. 2023). The gap in this guideline not only reflects the insufficient evidence of high‐quality dietary interventions, but also provides research space for exploring the application value of natural functional components such as plant flavonoids in PCOS assisted management.

### 1.1 Literature Search Strategy

This article is a narrative review that provides a comprehensive overview of evidence published between 2006 and 2026. A literature search was conducted using the keywords “flavonoids” and “polycystic ovary syndrome” in PubMed, Web of Science, and CNKI (China National Knowledge Infrastructure). Additional relevant articles were identified by manually screening the reference lists of retrieved papers and relevant reviews. The inclusion criteria were: (i) studies investigating the dietary sources, structural characteristics, or bioavailability of plant flavonoids; (ii) studies examining the molecular mechanisms of flavonoids in PCOS intervention; and (iii) clinical trials or observational studies evaluating the efficacy of flavonoid supplementation in PCOS patients. Studies not published in English or Chinese, or those unrelated to flavonoids or PCOS, were excluded. The representative literature was then organized and summarized according to the key themes of dietary sources, metabolism and bioavailability, molecular mechanisms, clinical evidence, and functional food development.

## 2 Dietary Sources, Structure, and Molecular Basis of Plant Flavonoids

Plant flavonoids are a large class of polyphenolic compounds derived from natural products. Due to their widespread distribution in the plant kingdom, they have attracted significant attention in various fields, including medicine and pharmacology (Mutha et al. 2021). They not only play an important role in diet and health but have also demonstrated multifaceted pharmacological activities in modern medical research. Generally speaking, plant flavonoids often possess antioxidant, anti‐inflammatory, and antitumor properties, as well as the ability to regulate immune function. In recent years, they have also demonstrated potential benefits in the management of female endocrine disorders, particularly polycystic ovary syndrome (PCOS). The following sections will systematically elaborate on the characteristics of plant flavonoids from four perspectives: dietary sources, classification by chemical structure, intestinal fate and bioavailability, and the structure–activity relationships of metabolic pathways related to PCOS.

### 2.1 Dietary Sources of Plant Flavonoids

**FIGURE 1:**
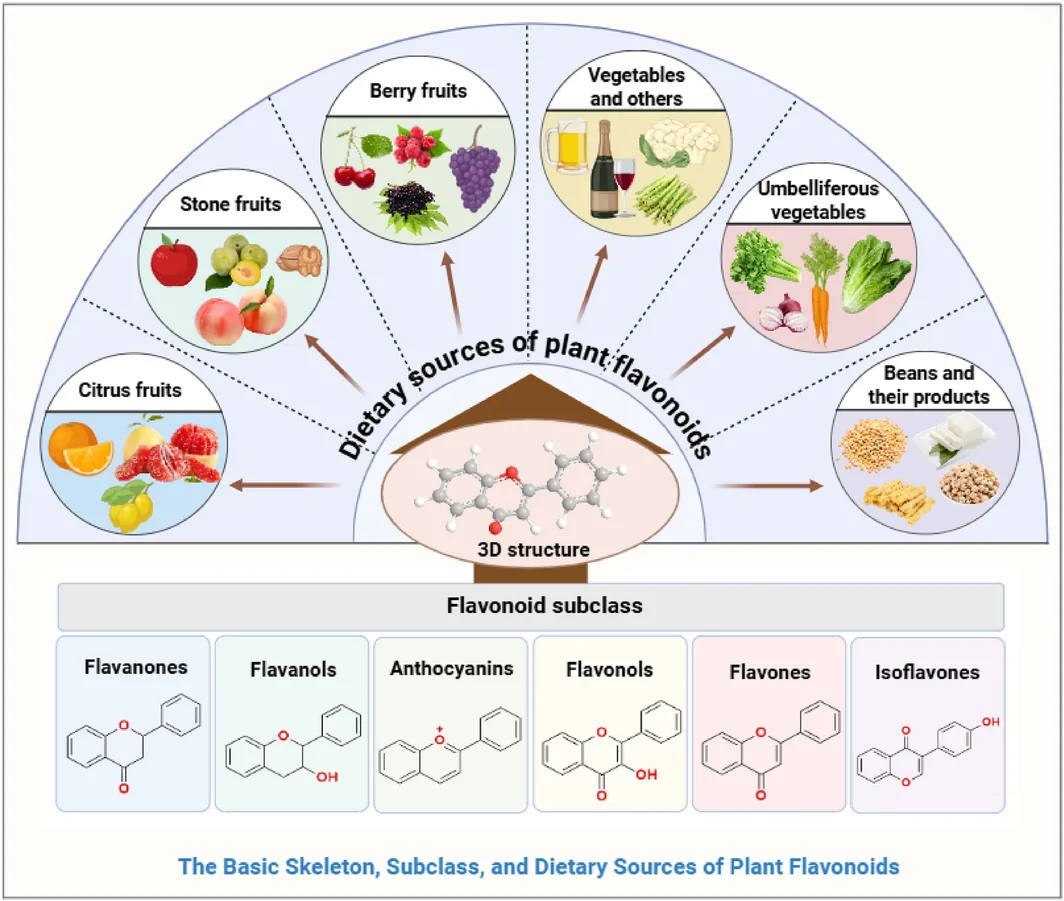
Major dietary sources of plant flavonoids by subclass. This schematic categorizes common foods and beverages rich in various flavonoid subclasses: Flavanones in citrus fruits, flavanols in apples and tea, anthocyanins in berries, and isoflavones in soybeans. It serves as a practical guide for designing flavonoid‐enhanced diets, emphasizing the diversity and accessibility of these bioactive compounds in everyday nutrition.

Plant flavonoids are not only important plant secondary metabolites but also indispensable bioactive components in the human diet; their intake is closely associated with the prevention of various chronic diseases and overall health (Galatro et al. 2024). Dietary flavonoids are extremely widespread in nature and serve as an important source of polyphenolic compounds in the daily diet. Dietary flavonoids possess a C15 basic skeleton (C6‐C3‐C6), and the arrangement of functional groups and hydroxyl groups on this skeleton determines their subclasses and specific biological functions. Based on their substituents, they can be classified into six distinct subclasses: flavones, flavonols, flavanones, flavanols, isoflavones, and anthocyanins (Zhao et al. 2024). They are primarily found in various plant‐based foods. Fruits are excellent sources of flavonoids; for example, citrus fruits (oranges, limes, lemons, and grapefruits) are rich in flavanones (such as naringin and naringenin); apples, peaches, pecans, plums, and cherries are rich in flavanols; berries (cherries, raspberries, elderberries, strawberries, grapes), red cabbage, tomatoes, and eggplants are rich in anthocyanins (Galatro et al. 2024). Among vegetables, onions, asparagus, broccoli, leeks, celery, and kale are common sources of flavanols, while umbelliferous vegetables such as carrots, parsley, and celery, as well as lettuce, olives, onions, chili peppers, and chamomile, are rich in flavonoids (such as apigenin) (Galatro et al. 2024). In addition, legumes and their products (particularly soybeans and chickpeas) are major dietary sources of isoflavones (such as genistein and daidzein); beverages such as tea (rich in catechins, quercetin, etc.), coffee, cocoa, and red wine also contribute significantly to flavonoid intake (Galatro et al. 2024). Herbs and spices such as parsley, thyme, and traditional Chinese medicinal herbs (e.g., *Eucommia ulmoides* and hawthorn) are also concentrated sources of flavonoid compounds (Alirezalu et al. 2018; Hussain et al. 2016; Justesen and Knuthsen 2001) (Figures 1 and 4A). A systematic understanding of the distribution patterns of different flavonoid subclasses in plant‐based foods and their stability during processing is a prerequisite for the scientific screening of functional food ingredients, the optimization of processing methods, and the precise design of dietary intervention programs.

### 2.2 Chemical Structural Classification of Plant Flavonoids

Based on differences in their parent ring structures and substituents, plant flavonoids can be classified into various types, including flavones, flavanols, flavanones, isoflavones, and chalcones (Armanini et al. 2022). These compounds are widely found in fruits, vegetables, flowers, tea leaves, and numerous medicinal plants, with flavones, flavanols, and isoflavones being among the more common subclasses.

In terms of representative compounds, total flavonoids from *Eucommia ulmoides* , fennel, and blue sunflower are relatively abundant in various traditional Chinese medicinal materials and herbal plants. Modern pharmacological studies indicate that these total flavonoids may comprehensively regulate the body's adaptive capacity regarding glucose and lipid metabolism and hormonal levels, thereby exerting a holistic regulatory effect on endocrine and metabolic imbalances. At the same time, luteolin and quercetin—as widely distributed monomeric flavonoids—have also been extensively studied for their prominent anti‐inflammatory, antioxidant, and immunomodulatory effects (Li et al. 2016; Zhang et al. 2026). Huang et al. found that luteolin normalizes the estrous cycle, improves ovarian morphology, and regulates hormone levels; it significantly inhibits insulin resistance (IR), upregulates PI3Kp85α and pAKT protein levels, enhances antioxidant activity, and restores the protein and mRNA expression of Nrf2 and its downstream genes Hmox1 and Nqo1 (Huang and Zhang 2021). Shah et al. reported that quercetin treatment can normalize the estrous cycle, regulate the LH/FSH ratio and estrogen levels, restore normal expression of related genes, and reduce cholesterol and other indicators; as it acts on the pituitary‐ovarian axis, it may hold promise for the treatment of PCOS in humans (Shah et al. 2023). These compounds often act concurrently through multiple signaling pathways, offering potential benefits in alleviating insulin resistance, hyperandrogenism, and local inflammatory states, thereby providing both experimental and clinical foundations for their application in complex endocrine disorders such as PCOS.

### 2.3 Intestinal Fate and Determinants of Bioavailability of Plant Flavonoids

The health effects of plant flavonoids in the human body depend largely on the efficiency of their digestion, release, metabolism, and absorption in the gastrointestinal tract—that is, their bioavailability. This process is extremely complex; it is influenced not only by the chemical structure of the flavonoids themselves (such as their degree of glycosylation) but is also closely related to the food matrix, processing methods, and the composition of an individual's gut microbiota. Most flavonoid molecules possess a benzopyranone skeleton, and the attachment of hydroxyl groups, sugar residues, or other substituents to different rings or side chains directly affects key parameters such as polarity, molecular weight, and solubility, which to a certain extent determine the level of bioavailability following oral intake (Kumar and Pandey 2013). Generally speaking, flavonoids with lower polarity, smaller molecular weight, and better lipophilicity are more easily absorbed through the intestinal or cell membranes. Studies have shown that flavonoids conforming to Lipinski's rules (molecular weight ≤ 500 Da, LogP ≤ 5, hydrogen bond donor/acceptor ratio ≤ 5/10) are more readily absorbed via passive diffusion and can even cross the blood–brain barrier (Xu et al. 2025). However, the gut microbiota undergoes terminal deglycosylation to produce phenolic acid metabolites, and CYP3A4‐mediated metabolism and conjugation reactions in the enterohepatic circulation further alter their bioavailability (Favari et al. 2024; Ibrahim et al. 2025).

From a toxicological and safety perspective, dietary plant flavonoids generally exhibit low toxicity; in particular, flavonoid compounds derived from common foods or traditional Chinese herbal medicines are relatively safe for use. Toxicological assessments show that the urinary excretion rates of dietary kaempferol and quercetin range from only 2.5% to 40%, and predictions using SwissADME and ProTox‐II are consistent with the Veber/Egan rule, indicating no mutagenicity or hepatotoxicity (Islam et al. 2025; Xu et al. 2024). However, it is precisely this limited absorption and extensive metabolic conversion that often make it difficult to replicate in vivo the significant activity observed in in vitro experiments. Therefore, for further clinical application, appropriate formulation design or improvements in dosage forms are still needed to overcome the challenges posed by the low water solubility and bioavailability of some flavonoids. This suggests that one of the key pathways to overcoming this bottleneck lies precisely in the application of food processing technologies—by pretreating flavonoids or their rich extracts using modern food engineering methods such as microencapsulation (Bińkowska et al. 2024), nano‐encapsulation (Teng et al. 2023), and liposomal carriers (Faridi Esfanjani et al. 2018), it is possible to effectively improve their solubility, enhance gastrointestinal stability, regulate release behavior, and even achieve targeted delivery to the small intestine (Lv et al. 2024). Consequently, to maximize their therapeutic efficacy and safety in clinical applications, the rational design of functional foods containing flavonoid compounds should continue to be explored in depth.

### 2.4 Structure–Activity Relationships Underlying the Regulation of PCOS‐Related Metabolic Pathways by Plant Flavonoids

Since PCOS involves multidimensional changes such as hyperandrogenism, insulin resistance, and chronic inflammation (Azziz et al. 2016), plant flavonoids—which possess multiple pharmacological effects—exhibit broad target coverage and diverse intervention pathways. The following are three potential regulatory mechanisms closely associated with PCOS:

Plant flavonoids can modulate local ovarian endocrine function by regulating hormone synthesis and secretion. For example, soy isoflavones reduce testosterone production in ovarian interstitial cells by inhibiting the activity of 3β‐hydroxysteroid dehydrogenase (3β‐HSD) and 17β‐hydroxysteroid dehydrogenase (17β‐HSD) (Raihanah et al. 2024). Some flavonoids can intervene in the development of hyperandrogenism by downregulating enzymes involved in androgen synthesis in theca cells (such as CYP17A1) or by reducing excessive luteinizing hormone (LH) secretion. The glycosidic ligand core of quercetin and the ortho‐dihydroxyl group on its B‐ring confer upon it a unique ability to bind to proteins such as PI3K. This binding effectively blocks the PI3K signaling pathway, reducing the expression of the CYP17A1 enzyme in ovarian theca cells and further inhibiting the conversion of progesterone to androgens, thereby counteracting the abnormal androgenic state in the ovaries (Luo et al. 2022). In addition, flavonoids can regulate the function of the hypothalamic–pituitary–ovarian axis. Flavonoid intervention can improve serum LH levels in patients with PCOS and normalize the LH/FSH ratio, thereby alleviating the inhibitory effect of hyperandrogenism on follicular development (Li et al. 2024). At the same time, certain plant flavonoids can enhance the enzymatic activity of granulosa cells in converting androgens to estrogens, thereby maintaining a healthier balance of sex hormones and providing a more favorable internal environment for subsequent follicular development and ovulation. This structure–activity relationship suggests that, when screening functional food ingredients for dietary intervention in PCOS, priority should be given to flavonoids with these structural characteristics.

Plant flavonoids possess anti‐inflammatory, antioxidant, and immune microenvironment‐modulating properties. For example, total fenugreek flavonoids (Rafieian et al. 2024), luteolin, and quercetin have all demonstrated significant anti‐inflammatory and antioxidant characteristics in multiple in vitro and in vivo experiments. In a mouse model of PCOS, total fenugreek flavonoids alleviated the progression of PCOS by inhibiting the TLR4/NF‐κB signaling pathway, thereby reducing the expression of pro‐inflammatory cytokines, chemokines, and other inflammation‐related molecules in ovarian tissue. In a randomized, double‐blind clinical trial, quercetin reduced the levels of inflammatory factors such as IL‐6 and tumor necrosis factor‐α (TNF‐α) in the serum of PCOS patients, thereby effectively alleviating the inflammatory response and significantly improving the ovarian inflammatory microenvironment (Luo et al. 2022). Hawthorn leaf flavonoids reduce the infiltration of macrophages into the ovaries by inhibiting the phosphorylation of p38 MAPK and STAT3, thereby lowering the expression of inflammatory cytokines such as IL‐6 and TNF‐α in ovarian tissue. This reduces the infiltration and adhesion of inflammatory cells in the ovaries and improves the inflammatory state associated with PCOS (Lian et al. 2016). These plant flavonoids can inhibit the activation of inflammatory signaling pathways such as NF‐κB and MAPK, reduce the secretion of cytokines such as TNF‐α and interleukin‐6, and minimize damage to tissue cells caused by free radicals or reactive oxygen species. For women with PCOS who are in a state of chronic low‐grade inflammation and oxidative stress, this may play a positive role in reducing local inflammatory responses and optimizing the metabolic environment of the ovaries and peripheral tissues.

**FIGURE 2:**
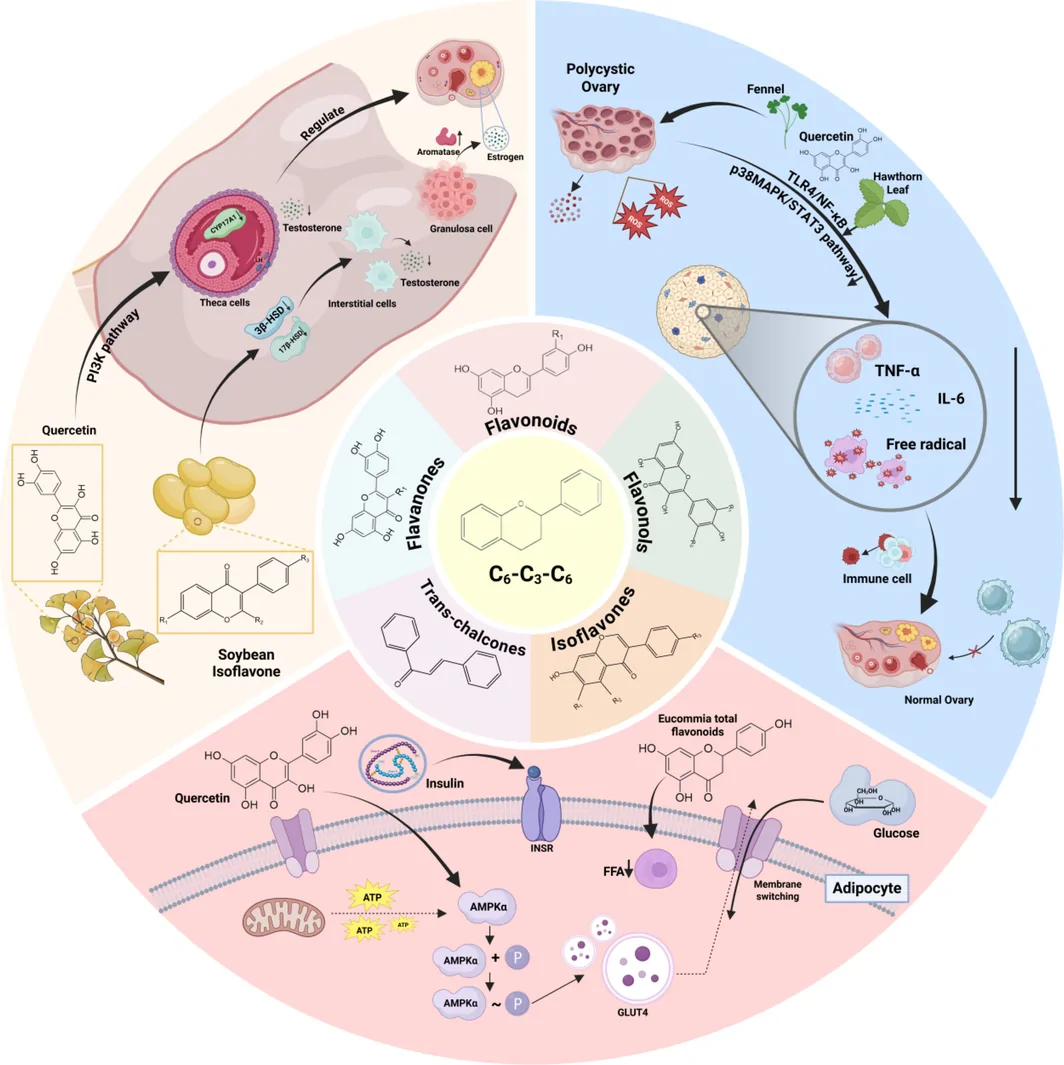
Basic skeleton, structural classification, and food‐relevant bioactivities of plant flavonoids. The figure maps the core C6‐C3‐C6 structure to major flavonoid subclasses (e.g., flavones, flavonols, isoflavones) and links them to key bioactivities pertinent to PCOS, including anti‐hyperandrogenic, anti‐inflammatory, antioxidant, and insulin‐sensitizing effects. This structure–activity relationship informs the targeted selection of flavonoid‐rich foods or extracts for functional food development.

Improving insulin resistance and glucose and lipid metabolism. Hyperinsulinemia and metabolic disorders, which frequently accompany PCOS, often form a vicious cycle that mutually reinforces hyperandrogenism. Evidence suggests that in adipocytes, quercetin promotes glucose uptake by activating the phosphorylation of the AMPKα subunit and facilitating the translocation of GLUT4 to the cell membrane. Clinical studies have shown that quercetin can downregulate the expression of the resistin gene in peripheral blood mononuclear cells and indirectly inhibit the expression of the luteinizing hormone receptor and insulin receptor genes; however, its effect on insulin resistance is not significant, possibly because the expression levels of the resistin gene and the administered concentration of quercetin were insufficient to effectively improve glucose metabolism (Rezvan et al. 2017). Other studies have indicated that total flavonoids from *Eucommia ulmoides* may have an activating or regulatory effect on lipid metabolic pathways. These signaling pathways are closely associated with reducing fat accumulation and lowering levels of free fatty acids and glycated hemoglobin, which may provide additional support for comprehensive metabolic control in patients with PCOS (Saikia et al. 2025). The aforementioned structure–activity relationships provide a molecular basis for understanding how plant flavonoids intervene in PCOS through dietary means (Figure 2); their specific signaling pathways and experimental evidence will be systematically discussed in Chapter 3.

## 3 Molecular Mechanisms and Experimental Studies on the Use of Plant Flavonoids for PCOS Intervention

**FIGURE 3:**
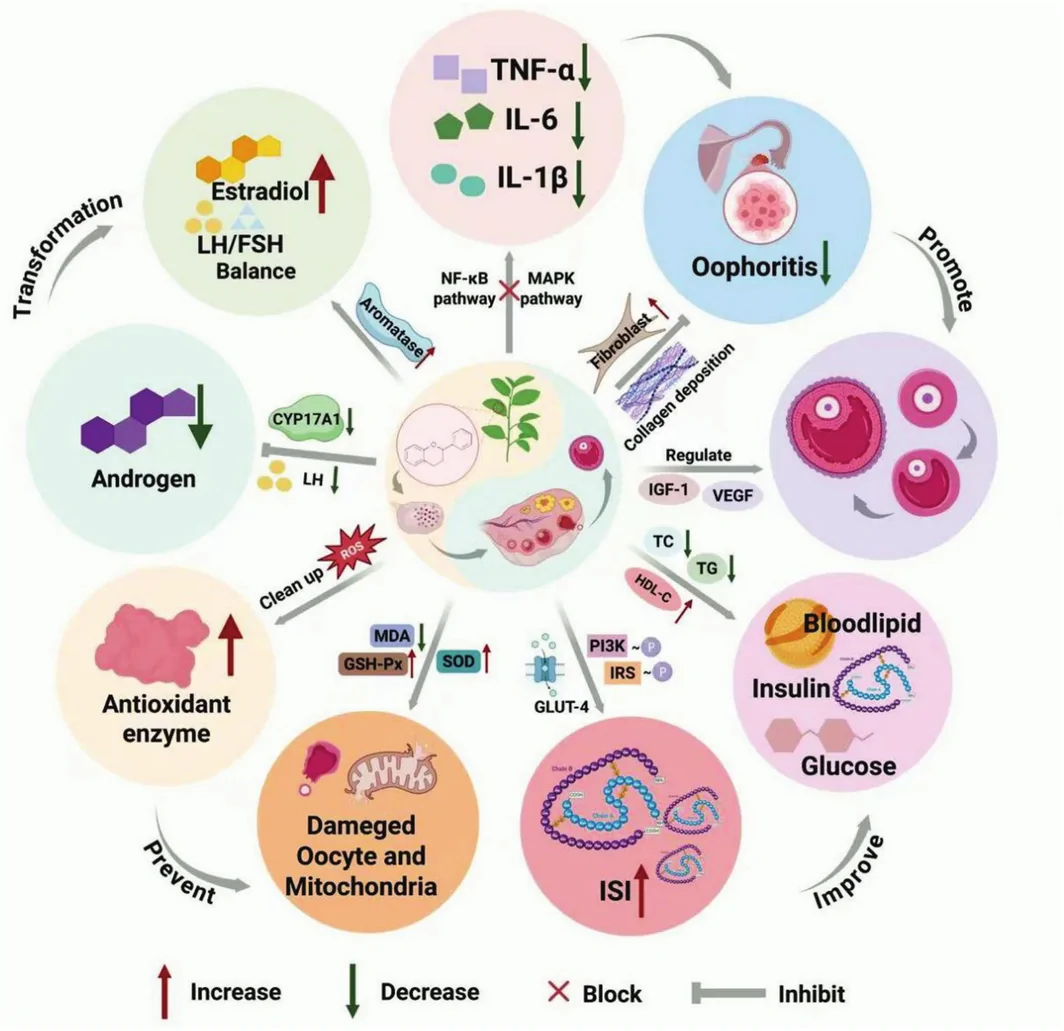
Multi‐target mechanisms of dietary flavonoids in ameliorating PCOS. The diagram synthesizes how food‐derived flavonoids intervene in PCOS pathology: Regulating sex hormones (reducing testosterone, balancing LH/FSH), improving insulin signaling and glucose metabolism, alleviating oxidative stress, and suppressing chronic inflammation. It visually integrates the “food as medicine” concept, demonstrating how a single dietary component can modulate a network of beneficial physiological responses.

As natural compounds with a wide range of sources and diverse mechanisms of action, plant flavonoids are gradually emerging as a potential new approach for PCOS intervention, thanks to their unique ability to regulate multiple targets. A wealth of research evidence indicates that plant flavonoids can play a positive role in multiple aspects, including endocrine regulation, anti‐inflammatory and antioxidant effects, improvement of insulin sensitivity, and protection of ovarian structure and function, and have yielded preliminary results in cellular and animal model studies (Armanini et al. 2022). Understanding these molecular mechanisms provides direct guidance for the development of functional foods: it helps researchers determine which flavonoid subclasses or structural features should be prioritized for enrichment, which signaling pathways can serve as biomarkers for the efficacy of dietary interventions, and which flavonoid combination strategies are appropriate for specific PCOS subtypes. Therefore, the following sections will systematically discuss the key molecular mechanisms and experimental research progress regarding the use of plant flavonoids for PCOS intervention (Figure 3).

### 3.1 Regulatory Effects on Endocrine Dysregulation

#### 3.1.1 Reduction of Androgen Levels (Testosterone, LH)

Hyperandrogenism is one of the most prominent endocrine abnormalities associated with PCOS (Dadachanji et al. 2018). Plant flavonoids may reduce testosterone levels in this process by inhibiting enzymes involved in androgen synthesis in theca cells (such as CYP17A1) or by interfering with the excessive secretion of luteinizing hormone (LH). Some in vitro studies suggest that specific flavonoids, such as quercetin, luteolin, and total flavonoids from *Eucommia ulmoides* , can directly or indirectly inhibit the activation of the LH receptor signaling pathway, thereby preventing excessive stimulation of androgen production in the ovaries (Peng et al. 2021). This intervention in the hyperandrogenic state provides an important foundation for alleviating clinical symptoms such as hirsutism and acne, as well as mitigating impaired follicular development.

#### 3.1.2 Increasing Estradiol Levels and Balancing the LH/FSH Ratio

The LH/FSH ratio is often significantly elevated in women with PCOS, whereas the normal ovulatory process requires FSH to stimulate granulosa cells to produce estradiol, thereby supporting the maturation and rupture of healthy follicles. Studies indicate that certain flavonoids can enhance aromatase activity in granulosa cells or upregulate its gene expression, thereby improving the efficiency of androgen‐to‐estrogen conversion in follicular tissue and consequently alleviating estradiol deficiency (Zhang et al. 2022). To a certain extent, this can balance the LH/FSH ratio, laying a solid foundation for stabilizing cyclic ovulation and endocrine rhythms.

### 3.2 Anti‐Inflammatory and Antioxidant Effects

#### 3.2.1 Inhibition of the Expression and Release of Inflammatory Cytokines (TNF‐α, IL‐6)

Chronic inflammation is considered one of the core pathological factors in PCOS, and numerous studies have focused on the regulatory effects of flavonoids on inflammatory signaling pathways (Tan et al. 2024). Mechanistically, flavonoids can attenuate the production and release of inflammatory cytokines such as TNF‐α, IL‐6, and interleukin‐1β (IL‐1β) by blocking the activation of key signaling pathways, including NF‐κB and MAPK (Liu et al. 2025; Lu et al. 2022). This not only reduces the negative impact of systemic inflammation on the body but also helps alleviate the local inflammatory microenvironment in the ovaries, protecting follicular cells from damage caused by persistent inflammation.

#### 3.2.2 Alleviating Oxidative Stress and Increasing Antioxidant Enzyme Levels

It is well known that PCOS is often accompanied by elevated oxidative stress, and the functions of endogenous antioxidant systems—such as superoxide dismutase (SOD) and glutathione peroxidase (GSH‐Px)—are also relatively impaired (Uçkan et al. 2022). Due to their polyphenolic structure, plant flavonoids can directly scavenge reactive oxygen species (ROS) or, by activating nuclear transcription factors such as Nrf2, induce the synthesis of more antioxidant enzymes, thereby enhancing the body's defense against oxidative damage. Practical studies have shown that supplementing PCOS model animals with specific flavonoids reduces MDA (malondialdehyde) levels in the body and generally enhances SOD and GSH‐Px activity to a certain extent, playing a positive role in preventing damage to key structures such as the oocyte plasma membrane and mitochondria (Darabi et al. 2020). Furthermore, some flavonoids exhibit direct mitochondrial protective effects. By maintaining the stability of the mitochondrial membrane potential, reducing mitochondrial reactive oxygen species (ROS) leakage, and regulating pathways related to mitochondrial biogenesis (such as PGC‐1α), they fundamentally alleviate oxidative stress and chronic inflammatory damage in the ovarian microenvironment of PCOS (Abdi et al. 2025; Hu et al. 2023).

### 3.3 Improving Insulin Sensitivity and Metabolic Abnormalities

#### 3.3.1 Regulating the Expression of Proteins Involved in Insulin Signaling Pathways

Hyperinsulinemia and insulin resistance are key pathophysiological mechanisms underlying PCOS (Moghetti 2016). Plant flavonoids can enhance the insulin response in peripheral tissues through various mechanisms, including promoting normal phosphorylation levels of signaling proteins such as insulin receptor substrate (IRS) and phosphoinositide 3‐kinase (PI3K), and may also upregulate the expression and translocation rate of glucose transporter (GLUT4). Some studies have also found that plant flavonoids activate the AMPK (adenylate‐activated protein kinase) pathway, thereby optimizing cellular utilization of glucose and fatty acids and improving overall insulin sensitivity (Wu et al. 2022).

#### 3.3.2 Reduction of Fasting Blood Glucose, Insulin Levels, and Lipid Parameters

Patients with PCOS often present with elevated fasting blood glucose and excessive or unstable insulin secretion, and their lipid profiles typically show elevated levels of triglycerides and low‐density lipoprotein cholesterol (LDL‐C) (Bates and Legro 2013). Relevant animal experiments and preclinical studies indicate that quercetin, total flavonoids from *Eucommia ulmoides* , and other compounds can significantly lower fasting blood glucose levels and serum insulin concentrations, while improving lipid parameters—such as reducing cholesterol or triglycerides and increasing high‐density lipoprotein (HDL‐C) (Lai et al. 2021). Regarding obesity‐related lipid metabolism disorders, flavonoids can further mitigate the cumulative negative effects of insulin resistance by reducing hepatic steatosis and adipocyte hypertrophy.

### 3.4 Protection of Ovarian Structure and Function

#### 3.4.1 Reducing Inflammation and Fibrosis in Ovarian Tissue

Chronic inflammation and tissue fibrosis often lead to damage to ovarian structure, preventing follicles from ovulating or maturing normally. The anti‐inflammatory properties of plant flavonoids are expected to block or delay the invasion of inflammatory factors into ovarian tissue, while simultaneously inhibiting excessive fibroblast proliferation and collagen deposition, thereby maintaining a healthier microstructure of the ovarian surface and follicular cavity (Al‐Khayri et al. 2022). As the degree of local inflammation and fibrosis decreases, the environment for follicular growth improves, which may increase the rate of potential ovulation.

#### 3.4.2 Promoting Normal Follicular Development and Ovulation

Once endocrine balance is restored, inflammation is alleviated, and insulin sensitivity improves, follicular development often returns to normal. Some animal studies have demonstrated that flavonoid intervention can significantly reduce the number of cystic follicles in the ovaries, increase the proportion of mature follicles and corpus luteum, and boost ovulation and conception rates (Shi et al. 2019). Some studies have also observed that plant flavonoids can regulate growth factors such as IGF‐1 and VEGF at key stages of follicular development, providing auxiliary support for the ovulation process jointly regulated by estrogen and FSH (Sair and Liu 2022).

The aforementioned mechanistic studies confirm the multi‐target regulatory capacity of plant flavonoids on pathological processes associated with polycystic ovary syndrome (PCOS); however, transforming these mechanistic “possibilities” into “effectiveness” on consumers' dining tables still requires overcoming engineering obstacles such as bioavailability, food matrix stability, and dietary compliance. The next chapter will systematically explore, from the practical perspective of functional food development, how to bridge these gaps through food processing technologies and formulation design.

## 4 Development of Functional Foods Containing Plant Flavonoids and Targeted Dietary Intervention Strategies

The potential value of plant flavonoids in PCOS animal models and in vitro experiments is becoming increasingly evident through mechanisms such as the regulation of endocrine hormones, the inhibition of inflammatory pathways, and the improvement of insulin signaling. However, whether dietary interventions with plant flavonoids can move from theory to the dinner table depends on the simultaneous establishment of three pillars: enhancing the bioavailability of flavonoids through food processing methods (Polia et al. 2022); ensuring their stability and sensory quality in food matrices through formulation design; and translating laboratory dosages into daily regimens that consumers can adhere to long‐term through dietary strategies based on nutritional principles. Accordingly, this chapter will analyze, from the practical perspective of functional food development, the research progress, technical approaches, and industrialization bottlenecks associated with each of these aspects, with the aim of providing a practical roadmap for the research, development, and commercialization of flavonoid‐based functional foods.

### 4.1 Implications of Clinical Evidence for Functional Food Development and the Gap Between Research and Practical Application

**FIGURE 4:**
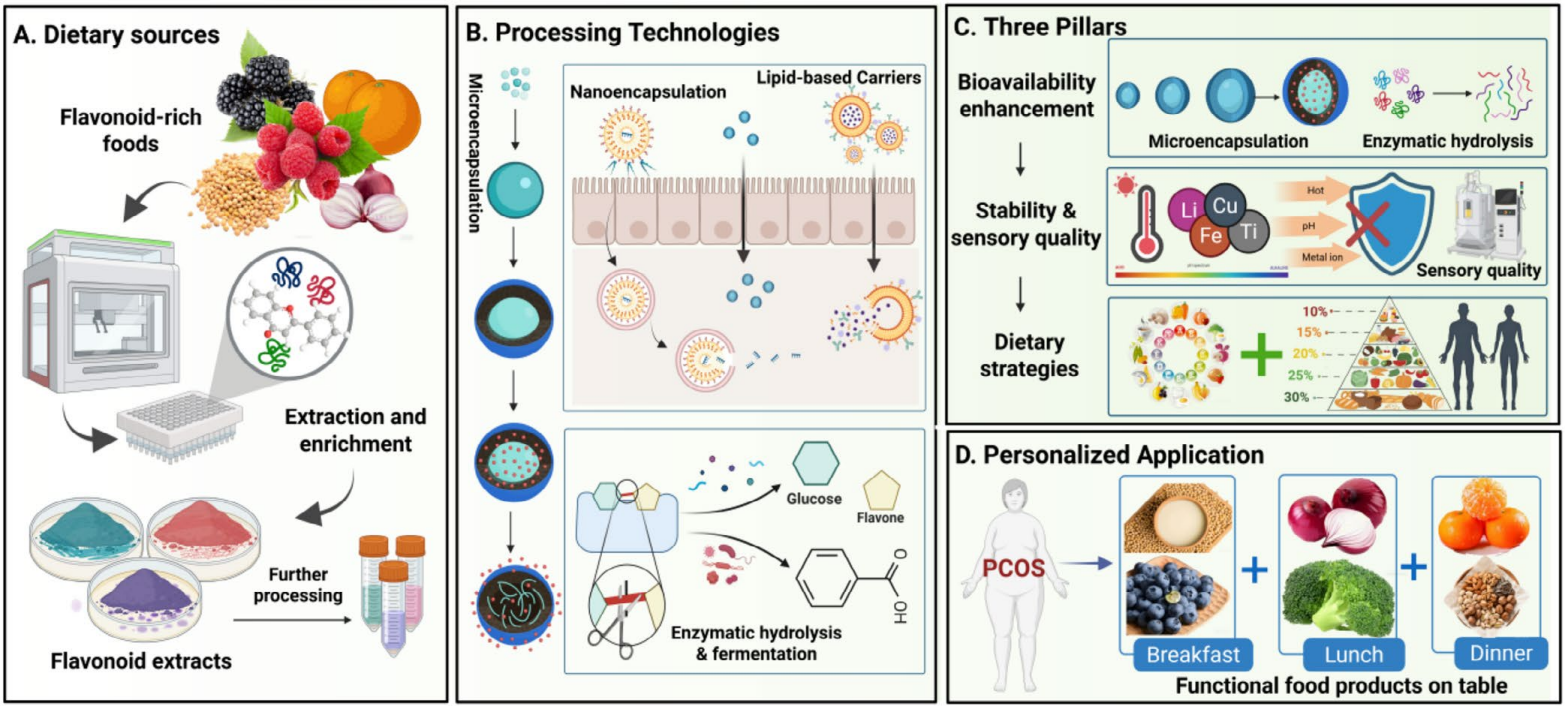
Translational pathway from dietary flavonoids to functional food products for PCOS management. This schematic illustrates the stepwise framework for developing flavonoid‐enriched functional foods for PCOS. (A) Dietary sources: Flavonoid‐rich foods (fruits, vegetables, soy, tea) serve as raw materials for flavonoid extracts. (B) Processing technologies: Nanoencapsulation, lipid‐based carriers, microencapsulation, enzymatic hydrolysis, fermentation, and extraction/enrichment are applied to enhance flavonoid bioavailability. (C) Three pillars: Bioavailability enhancement, stability and sensory quality control, and dietary strategies constitute the core of functional food development. (D) Personalized application: Tailored flavonoid formulations are translated into daily dietary plans (breakfast, lunch, dinner) as functional food products for different PCOS phenotypes. Abbreviation: PCOS, polycystic ovary syndrome.

In recent years, randomized controlled trials and meta‐analyses have preliminarily confirmed that plant flavonoid supplementation has certain adjunctive therapeutic effects for patients with PCOS, including reducing androgen levels, improving the insulin resistance index (HOMA‐IR), and downregulating inflammatory factors (TNF‐α, IL‐6) (Jiang et al. 2025; Luo et al. 2022; Rezvan et al. 2017). However, most of these clinical studies used high‐dose extracts or purified monomers, had short intervention periods (8–12 weeks), and involved a limited number of participants (Hossain et al. 2025; Jia et al. 2021). More critically, most intervention protocols administered flavonoids to participants in the form of “capsules,” rather than integrating them into daily diets or functional foods. While this method of administration helps validate the biological activity of flavonoids, it is severely disconnected from real‐world dietary scenarios and thus cannot provide direct evidence for the formulation design of functional foods, recommended daily intake levels, or consumption methods. Furthermore, the effective doses observed in clinical studies (typically several hundred milligrams of flavonoid aglycone equivalents per day) are far higher than typical daily dietary intake levels (averaging approximately 20–50 mg/day in Western populations) (Chen et al. 2022), This “dietary‐clinical dose gap” reveals the core contradiction in translating flavonoids from clinical validation to public dietary intervention—the issue is not “whether they are effective,” but “how to achieve effective exposure levels through food sources.” This raises three core scientific questions for the development of functional foods: (1) How can processing technologies overcome the inherent bottleneck of low oral bioavailability of flavonoids? (2) How can the stability and bioactivity of flavonoids be maintained in complex food matrices? (3) How can laboratory doses be translated into daily dietary regimens that are feasible and acceptable to consumers? The overall translational pathway from dietary sources to personalized functional food products is illustrated in Figure 4.

### 4.2 Food Processing Strategies to Enhance the Bioavailability of Plant Flavonoids

The generally low oral bioavailability of plant flavonoids is the greatest technical obstacle to transforming them from “active ingredients” into “effective foods.” Unlike drug development, where this issue is circumvented through chemical modification or injection, functional foods must address this challenge within the constraints of being “edible,” “processable,” and “cost‐effective.” In recent years, the field of food engineering has developed a series of mature physical, chemical, and biological processing methods that can significantly improve the gastrointestinal fate of flavonoids without altering their core molecular structure (Aatif 2023; Taldaev et al. 2025) (Figure 4B).

#### 4.2.1 Microencapsulation and Nano‐Encapsulation Technologies

Microencapsulation involves encapsulating flavonoid extracts within a matrix material (such as maltodextrin, cyclodextrin, gum arabic, whey protein, etc.) to form micron‐sized particles, thereby protecting them from degradation by light, oxygen, heat, and gastric acid, and enabling targeted release in the intestine (Lv et al. 2024; Teng et al. 2023). For flavonoid aglycones with low lipophilicity, cyclodextrin inclusion technology can significantly improve water solubility by encapsulating their nonpolar regions within hydrophobic cavities (Lima et al. 2019). Furthermore, nano‐encapsulation technologies (particle size < 200 nm), such as nano‐lipid carriers, nanoemulsions, and solid lipid nanoparticles, can enhance absorption rates through intercellular transport pathways in small intestinal epithelial cells. Studies have shown that when quercetin is encapsulated in nanostructured lipid carriers, its oral relative bioavailability increases 3.2‐fold compared to the parent compound, and its plasma antioxidant activity is significantly enhanced (Peralta‐Cuevas et al. 2025; Teng et al. 2023). From the perspective of the food industry, these technologies are feasible for large‐scale production and can be used to develop flavonoid‐fortified beverages, dairy products, and solid foods.

#### 4.2.2 Lipid Carriers and Micellar Delivery

Flavonol aglycones with good fat solubility (such as kaempferol and apigenin) can be incorporated into food‐grade lipid carriers (such as medium‐chain triglyceride microemulsions and phospholipid liposomes) to form mixed micelles in the gastrointestinal tract, thereby improving their dispersion in intestinal fluid and the efficiency of transport to the brush border of intestinal epithelial cells (Bilia et al. 2019; Lv et al. 2024). In addition, lipid excipients can inhibit the efflux of P‐glycoprotein in the small intestine, prolonging the retention time of flavonoids within intestinal epithelial cells (Nguyen et al. 2021). This strategy is particularly suitable for developing lipid‐containing functional foods (such as functional edible oils, dairy products, and spreads), achieving the dual function of “the carrier serving as the food matrix.”

#### 4.2.3 Enzymatic Hydrolysis and Fermentation Technologies

The vast majority of dietary flavonoids exist in the form of glycosides, which are too polar to readily cross cell membranes. By incorporating β‐glucosidase treatment into food processing (e.g., enzymatic hydrolysis of fruit juice, fermentation of soy milk), flavonoid glycosides can be hydrolyzed into aglycones, directly improving their absorption rate in the small intestine (Angelotti et al. 2020; Favari et al. 2024). Fermentation processes (such as lactic acid bacteria fermentation and yeast fermentation) not only achieve deglycosylation but also produce small‐molecule bioactive metabolites, such as phenolic acids, through microbial metabolism, thereby further enhancing biological activity (Alharbi 2026). Currently, products such as fermented soy milk and fermented tea (e.g., Pu'er tea) are successful commercial examples based on this principle, and their application in the development of functional foods holds broad prospects.

#### 4.2.4 Synergistic Enhancement Strategies for Food Components

Certain foods do not contain flavonoids themselves but can significantly influence their bioavailability. For example, small amounts of dietary lipids can promote the micellization of flavonoid aglycones (Gonzales et al. 2015); piperine (black pepper extract) can inhibit glucuronyltransferase (UGT) activity in the intestine and liver, thereby reducing first‐pass metabolism of flavonoids and increasing systemic exposure (Chen et al. 2022). In the formulation of functional foods, the inclusion of such “adjuvant factors” can enhance the bioavailability of flavonoids without increasing their dosage, offering multiple benefits such as cost‐effectiveness and improved palatability (Table 1).

**TABLE 1:** Comparison of food processing technologies for enhancing the bioavailability of plant flavonoids.

Technical strategy	Technical principles	Applicable flavonoid types	Advantages	Limitations	Commercialization readiness	References
Microencapsulation	The coating isolates the contents from the external environment, enabling targeted release in the intestine.	Various types of flavonoid extracts	The process is well‐established, relatively low‐cost, and provides stabilization benefits	Release efficiency is greatly influenced by the type of wall material	High (already widely used in the food industry)	Teng et al. 2023; Xiang et al. 2023
Nano‐encapsulation	Nanoparticle size promotes intercellular transport and lymphatic absorption	Fat‐soluble aglycones, flavonoid extracts	Bioavailability has increased significantly (more than threefold)	High equipment requirements and relatively high costs	In progress (laboratory‐proven, scaling up)	Ayala‐Fuentes and Chavez‐Santoscoy 2021; Taldaev et al. 2025
Liposome/Micelle carriers	Lipid carriers form mixed micelles, which improve dispersion in intestinal fluid.	Aglycones with good fat solubility	Both enhances absorption and masks the taste	Issues related to lipid oxidative stability	China (where applications in dairy products and beverages are relatively well‐established)	Morsy et al. 2025; Ranjbar et al. 2023
Enzymatic deglycosylation	β‐glucosidase hydrolyzes glycosides into aglycones	Flavonoid glycosides	Directly increases absorption in the small intestine	Enzymatic hydrolysis conditions must be controlled, as this may result in a bitter taste.	High (fermented foods are already used commercially)	Cunha et al. 2026; Bodakowska‐Boczniewicz and Garncarek 2025; Chen et al. 2022
Fermentation and conversion	Microbial metabolism enables deglycosylation and structural transformation	Various types of flavonoid glycosides	Conjointly produce active metabolites such as phenolic acids	Strain screening is complex, and there is significant batch‐to‐batch variation.	High (fermented soy milk, Pu'er tea, etc.)	Alharbi 2026; Yang et al. 2023
Synergy of auxiliary materials	Piperine inhibits UGT; lipids promote micellization	Various types of flavonoids	Simple recipe, low cost	The addition of ingredients may affect the flavor	High (can be used directly in product formulations)	Racz et al. 2022; Srinivasan 2007

### 4.3 Stability and Sensory Quality Control in the Formulation of Functional Foods

Flavonoids face two key challenges during food processing and storage: chemical instability and undesirable sensory characteristics. The former primarily manifests as oxidative degradation and structural rearrangement catalyzed by light, heat, pH, and metal ions (particularly in anthocyanins and flavonols); the latter is typically characterized by the inherent bitterness and astringency of flavonoids (Cao et al. 2021; Enaru et al. 2021; Laaksonen, n.d.). These factors directly limit the addition levels of flavonoids in conventional food systems and their consumer acceptance (Figure 4C).

#### 4.3.1 Strategies for Improving Processing and Storage Stability

Differentiated processing and preservation strategies must be developed based on the degradation patterns of different flavonoid subclasses. Anthocyanins are extremely sensitive to pH and rapidly fade and degrade under neutral and alkaline conditions; therefore, anthocyanin‐rich foods should be processed and stored in acidic systems (such as acidic beverages and fermented milk) (Cortez et al. 2017); Although flavanols are relatively heat‐stable, they are susceptible to enzymatic oxidation by polyphenol oxidase and must be controlled through color‐preserving measures such as blanching or the addition of ascorbic acid (Vámos‐Vigyázó 1981). Microencapsulation technology serves a dual purpose here—it not only enhances bioavailability but also protects flavanols from external environmental factors through the physical barrier provided by the encapsulation material, thereby extending the product's shelf life.

#### 4.3.2 Masking Bitterness and Optimizing Sensory Properties

Flavonoids typically exhibit varying degrees of bitterness and astringency, which is particularly noticeable in functional foods consumed directly, such as beverages and jellies (Lesschaeve and Noble 2005). Common masking strategies in the food industry include: cyclodextrin inclusion (masking bitter groups through encapsulation), adding sweeteners or flavor modifiers (such as luo han guo glycosides and vanillin) to balance the taste, and dispersing flavonoid components in a lipid matrix to reduce their direct exposure in the mouth (Goldberg et al. 2017). Furthermore, converting flavonoid glycosides into aglycones through fermentation can also alter the bitterness threshold to some extent. The optimization of sensory quality directly determines the market viability of functional foods and is an engineering dimension that cannot be overlooked in product development.

### 4.4 Practical Dietary Strategies and Personalized Nutrition Plans for Individuals With PCOS

Translating flavonoids from laboratory doses into everyday dietary interventions for the general public relies not only on food processing technologies but also on dietary guidance based on nutritional principles and personalized, tiered strategies. For women with PCOS, the most fundamental and safest strategy is to prioritize food combinations that are high in flavonoids and readily available at an affordable price in their daily diet. For example: for breakfast, soy milk (soy isoflavones) paired with blueberries (anthocyanins); for lunch, a cold onion salad (quercetin) paired with broccoli (kaempferol); replace coffee with green tea (catechins) in the afternoon; and consume citrus fruits (hesperidin) after dinner (Galatro et al. 2024). This “food combination method” not only enhances overall bioactivity through the synergistic effects of multiple flavonoids but also mitigates the potential risks associated with high‐dose supplementation of a single compound. Regarding food pairing techniques, it is recommended to consume flavonoid‐rich meals with moderate amounts of fat (such as olive oil or nuts) to utilize lipids in promoting the absorption of more fat‐soluble aglycones, or to pair meals with a small amount of black pepper to leverage the UGT‐inhibitory effect of piperine to increase systemic exposure to flavonoids (Gurley et al. 2012; Srinivasan 2007) (Figure 4D). The complete pathway from dietary sources to personalized dietary application is summarized in Figure 4, which integrates the three pillars of functional food development with phenotype‐based stratification.

## 5 Future Research Directions and Outlook

As mentioned earlier, fragmented clinical evidence, low bioavailability, and a lack of quality control standards are the three major bottlenecks hindering the development of flavonoid dietary interventions. Future research should address these challenges by seeking breakthroughs in areas such as the targeted design of functional foods empowered by multi‐omics and artificial intelligence (AI) technologies, the establishment of dietary compliance assessment systems for high‐quality clinical studies, and precision dietary stratification strategies based on individual metabolic phenotypes. Through interdisciplinary collaboration and technological innovation, we can provide more comprehensive and scientifically sound solutions for plant flavonoid interventions in PCOS, driving the evolution of plant flavonoid‐based functional foods from “one‐size‐fits‐all products” to “customizable solutions.”

### 5.1 Multi‐Omics and AI Technologies Empowering Targeted Design of Functional Foods

PCOS involves multiple signaling pathways and phenotypic characteristics, and single‐dimensional research often fails to capture its complex pathological network (Sikiru et al. 2023). In the future, by integrating multi‐omics technologies—including genomics, proteomics, metabolomics, and even transcriptomics and epigenetics (Elfiky et al. 2025; Kahfi et al. 2025) —we can conduct a comprehensive analysis of the key targets and mechanisms of action of plant flavonoids in PCOS intervention. This approach will not only provide insights into how flavonoids act across multiple pathways—including the hypothalamic–pituitary–ovarian axis, inflammatory pathways, and insulin sensitivity—but, more importantly, will provide molecular‐level evidence for the “targeted formulation” of functional foods—that is, identifying which combinations of flavonoids hold the greatest potential for intervention in specific PCOS subtypes.

The introduction of artificial intelligence (AI) and big data platforms is expected to significantly enhance the efficiency of drug screening and the identification of synergistic targets (Xu et al. 2026). The use of AI algorithms can help researchers predict the potential effects of different flavonoid molecules on multiple signaling pathways (Sun et al. 2026) and, by taking into account differences among patient populations, screen for core compounds or compound combinations that are more specifically targeted to the characteristics of this disease, thereby avoiding potential antagonistic effects or the risk of adverse reactions, and providing actionable, visual guidance for multidimensional PCOS treatment. Furthermore, AI technology can be used to analyze large‐scale dietary survey data (Cohen et al. 2023), uncovering associations between flavonoid intake patterns and PCOS risk across different regions and dietary cultures, thereby providing data support for the development of regional functional foods.

### 5.2 High‐Quality Clinical Research and Assessment of Dietary Adherence

Although some clinical evidence has been accumulated regarding the use of plant flavonoids in the treatment of polycystic ovary syndrome (PCOS), the overall quality of existing studies remains insufficient to support regulatory approval of functional foods and health claims. Currently, the vast majority of randomized controlled trials (RCTs) suffer from limitations such as insufficient sample size, overly short observation periods, single‐center designs, and lack standardized dietary background controls (Hossain et al. 2025). There is an urgent need to establish a cross‐regional, multidisciplinary research network to conduct large‐scale, multicenter, randomized, double‐blind controlled trials (Malik et al. 2023), thereby more objectively reflecting the true efficacy of plant flavonoid interventions in different populations with polycystic ovary syndrome (PCOS).

More critically, future clinical study designs should align with the actual consumption scenarios of functional foods, specifically incorporating improvements on three levels. On the one hand, standardized dietary background controls should be introduced (Lamport 2012), involving baseline recording and continuous monitoring of participants' daily flavonoid intake through dietary frequency questionnaires or biomarkers to quantify and eliminate confounding effects from background diets; second, adherence assessment should be established as one of the core outcome measures. Adherence assessment tools tailored to functional foods (such as food diaries or smart packaging logs) should be developed, and adherence rates should be incorporated into analyses as moderator variables for intervention effects (Lucey et al. 2016); finally, establish a unified system of efficacy assessment indicators and a long‐term follow‐up mechanism; develop international evaluation standards for core indicators such as endocrine hormones, insulin resistance indices, and inflammatory markers; and extend the follow‐up period to 12 months or longer to assess the impact of flavonoid dietary interventions on reproductive outcomes and long‐term metabolic risks in patients with polycystic ovary syndrome (PCOS) (Moka et al. 2025).

### 5.3 Personalized Dietary Stratification Strategies Based on Metabolic Phenotypes

Patients with PCOS exhibit significant individual differences in terms of the degree of insulin resistance, obesity status, hyperandrogenism, and inflammatory phenotypes, making it difficult for “one‐size‐fits‐all” dietary intervention strategies to achieve optimal results in all patients (Farrell and Antoni 2010). Future research should focus on developing differentiated flavonoid dietary intervention plans based on individual metabolic phenotype characteristics, thereby achieving a transition from “population‐average effects” to “optimal individual matching.”

Specifically, the development of such a stratified strategy can follow the logic outlined below. First, identify the metabolic vulnerabilities of different PCOS subtypes. For patients primarily characterized by insulin resistance and obesity, prioritize formulations rich in quercetin and EGCG (which significantly activate AMPK (Wu et al. 2022)); for patients with prominent hyperandrogenism, prioritize formulations rich in soy isoflavones (anti‐androgenic effects (Raihanah et al. 2024)); and for the chronic inflammation phenotype, prioritize ingredients with prominent antioxidant activity, such as anthocyanins and hawthorn leaf flavonoids (Al‐Khayri et al. 2022). Second, integrate genomic and gut microbiota information to achieve precise matching. Individual differences in flavonoid absorption, metabolic conversion, and target sensitivity are influenced by polymorphisms in metabolic enzyme genes such as UGT, COMT, and CYP450, as well as by gut microbiota composition (Ahmadi et al. 2026; Cassidy and Minihane 2017). In the future, by analyzing an individual's key genotypes and gut microbiota characteristics, it will be possible to predict their metabolic efficiency and response intensity to different flavonoids. Finally, incorporate stratification strategies into the product design of functional foods. By designing a “modular” functional food matrix—introducing multiple formula products within the same product line tailored to different phenotypes—and using digital tools to recommend the most suitable product combinations to consumers, personalized nutrition can be transformed from an academic concept into a viable business model. It should be noted that the aforementioned stratification strategies are still based on logical deductions from existing mechanistic studies; their efficacy and safety still need to be validated through rigorously designed population‐based intervention trials.

## 6 Conclusion

Based on recent research advances, it has become increasingly recognized that plant flavonoids—a class of natural bioactive compounds found in a wide variety of sources—possess unique multi‐target synergistic advantages in regulating sex hormone secretion, improving insulin sensitivity, and providing anti‐inflammatory and antioxidant effects, as well as protecting ovarian function. This has laid a solid theoretical foundation for their application in the adjunctive management of PCOS. Extensive research evidence indicates that plant flavonoids can effectively reduce circulating testosterone levels and optimize the LH/FSH ratio by regulating androgen synthase activity and the function of the hypothalamic–pituitary‐ovarian axis; by activating the Nrf2 antioxidant pathway and inhibiting inflammatory signaling cascades such as NF‐κB and MAPK, they alleviate oxidative stress and chronic low‐grade inflammation; and by improving insulin signaling and correcting lipid metabolism disorders, they break the vicious cycle of mutual reinforcement between hyperinsulinemia and hyperandrogenism, thereby creating a suitable metabolic environment for normal follicular development and the restoration of ovulation.

However, a significant technical gap remains between “experimental efficacy” and “practical application”. The effective doses observed in clinical studies are far higher than those found in daily dietary intake, and plant flavonoids generally face the inherent bottleneck of low oral bioavailability, which constitutes a core obstacle to their translation from laboratory evidence into functional food products. From the practical perspective of functional food development, this review systematically summarizes the application progress of modern food processing technologies—such as microencapsulation, nano‐encapsulation, liposomal carriers, enzymatic hydrolysis and fermentation, and synergistic use of excipients—in enhancing the bioavailability of flavonoids. It analyzes key technical approaches for regulating stability and sensory quality in functional food formulation design and proposes practical dietary strategies and personalized nutritional plans tailored to different PCOS metabolic phenotypes.

Interdisciplinary technological integration and collaborative innovation among industry, academia, and research institutions will be the inevitable path to advancing plant flavonoids from academic papers to the dinner table and from mechanistic research to commercial products. The core task for the future lies in deeply integrating food processing technologies with the principles of nutritional science. While ensuring the stability, safety, and sensory quality of flavonoid‐based functional foods, the goal is to translate effective doses into daily dietary plans that consumers can adhere to over the long term, ultimately achieving the precision nutrition intervention objective of “promoting health through diet.”

## Author Contributions

**Jianding Wang:** conceptualization, investigation, visualization, writing – original draft. **Hejian Zhu:** conceptualization, investigation, writing – original draft, software. **Qing Shen:** conceptualization, visualization, supervision, writing – review and editing. **Yinghua Zhong:** writing – original draft, investigation. **Yunli Du:** investigation, data curation. **Wenxi Yu:** methodology, resources. **Fang Mao:** supervision, resources. **Zhitao Yao:** supervision, methodology. **Weiwei Zhu:** writing – original draft, visualization. **Lirong Sun:** data curation, visualization. **Qiu Lin:** conceptualization, supervision, writing – review and editing. **Xiaomin Xu:** conceptualization, writing – review and editing. **Jiong Zhu:** writing – original draft, validation, visualization.

## Funding

This work was supported by the Scientific Research Project in the Field of Agriculture and Social Development of Linping District under Grant 202303020017; the Medical and Health Science and Technology Project of Linping District under Grants LPWJ2022‐01‐08 and LPWJ‐02‐47; the Traditional Chinese Medicine Science and Technology Project of Zhejiang Province under Grants 2025ZX009, 2025ZX114, 2026ZL0694, and 2025ZX115; the Health Science and Technology Project of Hangzhou City under Grants B20230389 and B20241891; and the Science and Technology Key Project of Quzhou City under Grant 2024K082.

## Conflicts of Interest

The authors declare no conflicts of interest.

## Data Availability

No data were generated or analyzed in this study.
